# Supplementary material for: The Prognostic Value of Lymph Node Involvement after Neoadjuvant Chemotherapy Is Different among Breast Cancer Subtypes
Source: Cancers (Basel). 2021 Jan 6;13(2):171. doi: 10.3390/cancers13020171 (PMC7825348; doi:10.3390/cancers13020171)
Supplement: Supplementary file 1 [file cancers-13-00171-s001.pdf]

cancers

MDPI

Article

The Prognostic Value of Lymph Node Involvement after Neoadjuvant Chemotherapy Is Different among Breast Cancer Sub-Types

Lucie Laot [†,1], Enora Laas [†,1], Noemie Girard [1], Elise Dumas [2], Eric Daoud [2], Beatriz Grandal [2], Jean-Yves Pierga [3], Florence Coussy [3], Youlia Kirova [4], Elsy El Alam [5], Guillaume Bataillon [5], Marick Lae [6], Florence Llouquet [1], Fabien Reyal [1,2,*] and Anne-Sophie Hamy [2]

Table S1. Post-NAC nodal involvement according to NAC regimen among HER-2 positive BC patients (n=295).

		NAC with trastuzumab	NAC without trastuzumab	*p*
n =		204 (69.2%)	91 (30.8%)	
Post NAC node involvement	Negative	158 (77.5%)	47 (51.6%)	<0.00001
	Positive	46 (22.5%)	44 (48.4%)	

Abbreviations: NAC=neoadjuvant chemotherapy.

Table S2. Association between relapse free survival and pCR versus nodal status after neoadjuvant chemotherapy (Whole population, Luminal BC, TNBC and HER-2 positive BC).

		Whole population					Luminal BC				
**Variable**	**Class**	**Number**	**Events**	**HR**	**CI**	***p***	**Number**	**Events**	**HR**	**CI**	***p***
pCR	pCR	285	39	1			33	5	1		
	No pCR	911	332	2.87	[2.06 - 4]	<0.001	493	179	2.16	[0.89 - 5.24]	0.09
Nodal status	Negative	682	144	1			197	49	1		
	Positive	515	227	2.26	[1.83 - 2.78]	<0.001	329	135	1.7	[1.22 - 2.35]	0.002
		**TNBC**					***HER*-2 positive BC**				
**Variable**	**Class**	**Number**	**Events**	**HR**	**CI**	***p***	**Number**	**Events**	**HR**	**CI**	***p***
pCR	pCR	139	20	1			113	14	1		
	No pCR	236	96	3.48	[2.15 - 5.64]	<0.001	182	57	2.82	[1.57 - 5.06]	<0.001
Nodal status	Negative	280	60	1			205	35	1		
	Positive	96	56	3.81	[2.65 - 5.5]	<0.001	90	36	2.69	[1.69 - 4.29]	<0.001

Abbreviations: pCR=pathological complete response.

Table S3. Association between clinical and pathological pre and post-NAC parameters with relapse-free survival (Luminal BC population, univariate and multivariate analysis).

Luminal										
				Univariate				Multivariate		
**Variable**	**Class**	**Number**	**Events**	**HR**	**CI**	**p***	***p***	**HR**	**CI**	**p**
Pre-NAC parameters										
Age	[0 -50)	304	101	1			0.345			
	[50 -60)	158	56	1.06	[0.76 - 1.46]					
	60+	64	27	1.37	[0.9 - 2.1]					
Menopausal status	Pre	338	111	1			0.212			
	Post	184	71	1.21	[0.9 - 1.63]					
BMI	18.5-24.9	289	91	1			0.053			
	<18.5	24	10	1.63	[0.85 - 3.14]	0.141				
	25-29.9	135	46	1.08	[0.76 - 1.54]	0.682				
	>=30	75	36	1.64	[1.11 - 2.41]	0.012				

Smoking status	No	297	107	1			0.952			
	Yes	113	38	1.01	[0.7 - 1.46]					
BRCA mutation genes	BRCA1	8	2	1			0.997			
	BRCA2	6	2	1.24	[0.17 - 8.82]					
	others	1	0							
	No	74	24	1.16	[0.27 - 4.91]					
Clinical T stage (TNM)	T0-T1	21	10	1			0.01			
	T2	358	111	0.59	[0.31 - 1.14]	0.115				
	T3-T4	147	63	0.93	[0.48 - 1.81]	0.824				
Clinical N stage (TNM)	N0	235	75	1			0.1			
	N1-N2-N3	290	109	1.28	[0.95 - 1.72]					
Histological type	NST	444	150	1			0.08			
	Others	65	28	1.43	[0.96 - 2.15]					
KI67	[0-10)	52	17	1			0.778			
	[10-20)	79	29	1.13	[0.62 - 2.05]					
	>=20	157	58	1.21	[0.71 - 2.08]					
SBR grade	Grade I-II	324	119	1			0.643			
	Grade III	182	58	0.93	[0.68 - 1.27]					
LVI	No	144	59	1			0.162			
	Yes	84	27	0.72	[0.46 - 1.14]					
DCIS component	No	186	59	1			0.677			
	Yes	224	81	1.07	[0.77 - 1.51]					
CT regimen (NAC)	anthra-taxans	340	108	1			0.272			
	anthra	133	54	1.05	[0.75 - 1.46]					
	taxanes	8	1	0.37	[0.05 - 2.64]					
	others	45	21	1.49	[0.93 - 2.38]					
Post-NAC parameters										
pCR	No pCR	493	179	1			0.09			
	pCR	33	5	0.46	[0.19 - 1.13]	0.09				
Post-NAC LVI	No	237	75	1			0.046			
	Yes	189	78	1.38	[1.01 - 1.9]	0.046				
ypN	0	197	49	1			<0.001	1		
	[1-3]	216	70	1.24	[0.86 - 1.79]	0.245		1.18	[0.82 - 1.71]	0.377
	4 and more	113	65	2.8	[1.93 - 4.06]	<0.001		2.68	[1.84 - 3.89]	<0.001
RCB class	RCB-0	11	3	1			0.172			
	RCB-I	18	2	0.41	[0.07 - 2.43]					
	RCB-II	109	33	1.12	[0.34 - 3.64]					
	RCB-III	84	36	1.58	[0.48 - 5.13]					

p represents the p-value for the Wald test, and p* represents the individual p-value versus reference class. Abbreviations: pCR=pathological complete response; BMI=body mass index ; NST= no special type ; ER=estrogen receptor; PR=progesterone receptor; NAC=neoadjuvant chemotherapy; AC=anthracyclines; TILs=tumor infiltrating lymphocytes; RCB=residual cancer burden; LVI=lymphovascular invasion.

Table S4. Association between clinical and pathological pre and post-NAC parameters with relapse-free survival (TNBC population, univariate and multivariate analysis).

TNBC										
				Univariate				Multivariate		
**Variable**	**Class**	**Number**	**Events**	**HR**	**CI**	**p***	***p***	**HR**	**CI**	**p**
Pre-NAC parameters										
Age	[0 -50)	205	60	1			0.723			
	[50 -60)	115	38	1.17	[0.78 - 1.77]					
	60+	56	18	1.13	[0.67 - 1.91]					

Menopausal status	Pre	229	70	1			0.991			
	Post	143	44	1	[0.69 - 1.46]					
BMI	18.5-24.9	211	65	1			0.238			
	<18.5	12	1	0.22	[0.03 - 1.58]					
	25-29.9	103	31	1.01	[0.66 - 1.54]					
	>=30	49	19	1.4	[0.84 - 2.33]					
Smoking status	No	238	73	1			0.565			
	Yes	66	18	0.86	[0.51 - 1.44]					
BRCA mutation genes	BRCA1	22	6	1			0.673			
	BRCA2	5	2	1.43	[0.29 - 7.1]					
	No	83	18	0.79	[0.31 - 1.98]					
Clinical T stage (TNM)	T0-T1	31	7	1			0.001	1		
	T2	243	65	1.23	[0.56 - 2.69]	0.6		1.28	[0.58 - 2.8]	0.544
	T3-T4	102	44	2.42	[1.09 - 5.38]	0.03		2.29	[1.03 - 5.09]	**0.043**
Clinical N stage (TNM)	N0	171	49	1			0.282			
	N1-N2-N3	205	67	1.22	[0.85 - 1.77]					
Histological type	NST	340	101	1			0.367			
	Others	6	1	0.4	[0.06 - 2.9]					
KI67	[0-10)	10	2	1			0.558			
	[10-20)	16	6	2.18	[0.44 - 10.81]					
	>=20	141	52	2.18	[0.53 - 8.93]					
SBR grade	Grade I-II	53	17	1			0.917			
	Grade III	314	97	1.03	[0.61 - 1.72]					
LVI	No	82	28	1			0.121			
	Yes	38	18	1.6	[0.88 - 2.9]					
DCIS component	No	282	86	1			0.219			
	Yes	51	21	1.35	[0.84 - 2.18]					
CT regimen (NAC)	anthra-taxanes	287	86	1			0.728			
	anthra	63	24	1.2	[0.76 - 1.89]					
	taxanes	1	0							
	others	24	6	0.73	[0.32 - 1.69]					
Post-NAC parameters										
pCR	No pCR	236	96	1			<0.001			
	pCR	139	20	0.29	[0.18 - 0.47]	<0.001				
Post-NAC LVI	No	170	48	1			<0.001			
	Yes	64	38	2.65	[1.73 - 4.06]	<0.001				
ypN	0	280	60	1			<0.001	1		
	[1-3]	55	29	3.19	[2.05 - 4.98]	<0.001		3.17	[2.03 - 4.95]	**<0.001**
	4 and more	41	27	4.83	[3.06 - 7.63]	<0.001		4.52	[2.85 - 7.17]	**<0.001**

RCB class	RCB-0	123	18	1			<0.001
	RCB-I	23	4	1.22	[0.41 - 3.61]	0.717	
	RCB-II	131	50	3.09	[1.8 - 5.29]	<0.001	
	RCB-III	42	30	9.16	[5.08 - 16.54]	<0.001	

p represents the p-value for the Wald test, and p* represents the individual p-value versus reference class. Abbreviations: pCR=pathological complete response; BMI=body mass index ; NST= no special type ; ER=estrogen receptor; PR=progesterone receptor; NAC=neoadjuvant chemotherapy; AC=anthracyclines; TILs=tumor infiltrating lymphocytes; RCB=residual cancer burden; LVI=lymphovascular invasion.

Table S5. Association between clinical and pathological pre and post-NAC parameters with relapse-free survival (*HER-2* BC population, univariate and multivariate analysis).

HER2-positive										
				**Univariate**				**Multivariate**		
**Variable**	**Class**	**Number**	**Events**	**HR**	**CI**	**p***	***p***	**HR**	**CI**	**p**
Pre-NAC parameters										
**Age**	[0 -50)	169	49	1			0.066			
	[50 -60)	79	12	0.48	[0.25 - 0.9]	**0.022**				
	60+	47	10	0.74	[0.37 - 1.46]	0.386				
**Menopausal status**	Pre	179	51	1			**0.039**	1		
	Post	115	20	0.58	[0.35 - 0.97]	**0.039**		0.44	[0.23 - 0.84]	**0.013**
**BMI**	18.5-24.9	180	37	1			0.253			
	<18.5	12	4	1.74	[0.62 - 4.89]					
	25-29.9	66	19	1.52	[0.87 - 2.64]					
	>=30	35	11	1.71	[0.87 - 3.37]					
**Smoking status**	No	186	41	1			0.563			
	Yes	54	14	1.2	[0.65 - 2.2]					
**BRCA mutation genes**	BRCA1	1	1	1			0.423			
	BRCA2	3	0	0	[0 - Inf]					
	No	63	17	0.26	[0.03 - 1.96]					
**Clinical T stage (TNM)**	T0-T1	18	1	1			0.167			
	T2	196	47	4.69	[0.65 - 34]	0.126				
	T3-T4	80	22	6.06	[0.82 - 44.95]	0.078				
**Clinical N stage (TNM)**	N0	119	24	1			0.208			
	N1-N2-N3	176	47	1.37	[0.84 - 2.24]					
**Histological type**	NST	276	66	1			0.645			
	Others	3	1	1.59	[0.22 - 11.48]					
**KI67**	[0-10)	3	2	1			0.244			
	[10-20)	15	3	0.22	[0.04 - 1.3]					
	>=20	108	34	0.42	[0.1 - 1.76]					
**SBR grade**	Grade I-II	100	34	1			**0.006**	1		
	Grade III	182	33	0.51	[0.32 - 0.83]	**0.006**		0.39	[0.23 - 0.68]	**0.001**
**LVI**	No	41	11	1			0.085			
	Yes	49	21	1.91	[0.91 - 3.99]	0.085				
**DCIS component**	No	136	20	1			**0.006**			

	Yes	114	33	2.19	[1.25 - 3.82]	**0.006**	
**CT regimen (NAC)**	anthra-taxanes	218	40	1			**<0.001**
	anthra	39	19	3.2	[1.85 - 5.54]	**<0.001**	
	taxanes	16	3	1.12	[0.35 - 3.63]	0.848	
	others	22	9	2.64	[1.28 - 5.45]	**0.009**	
Post-NAC parameters							
**pCR**	No pCR	182	57	1			**<0.001**
	pCR	113	14	0.35	[0.2 - 0.64]	**<0.001**	
**Post-NAC LVI**	No	124	20	1			**<0.001**
	Yes	49	28	4.93	[2.77 - 8.78]	**<0.001**	
**ypN**	0	205	35	1			**<0.001**
	[1-3]	70	28	2.7	[1.64 - 4.43]	**<0.001**	
	4 and more	20	8	2.69	[1.24 - 5.8]	**0.012**	
**RCB class**	RCB-0	68	2	1			**0.002**
	RCB-I	24	1	1.52	[0.14 - 16.78]	0.732	
	RCB-II	69	19	10.35	[2.41 - 44.46]	**0.002**	
	RCB-III	15	6	16.53	[3.33 - 81.99]	**<0.001**	

p represents the p-value for the Wald test, and p* represents the individual p-value versus reference class. Abbreviations: pCR=pathological complete response; BMI=body mass index ; NST= no special type ; ER=estrogen receptor; PR=progesterone receptor; NAC=neoadjuvant chemotherapy; AC=anthracyclines; TILs=tumor infiltrating lymphocytes; RCB=residual cancer burden; LVI=lymphovascular invasion.

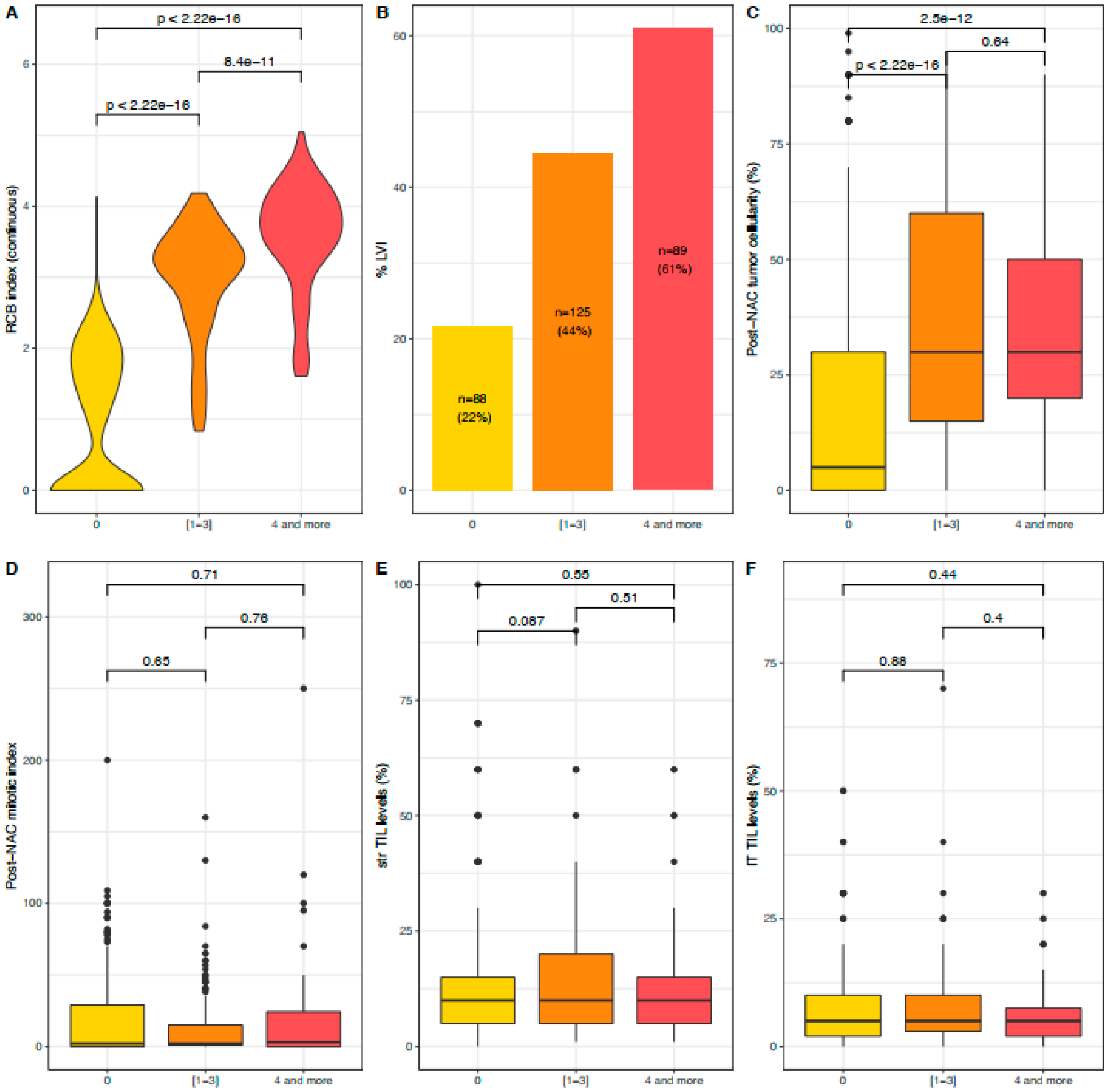

Figure S1. Association between post-NAC involvement and tumor characteristics in the whole population: RCB index (A); Lymphovascular invasion (B); Tumor cellularity (C); Post-NAC mitotic index (D); Stromal TIL levels (E). Intra tumoral (IT) TIL levels (F).

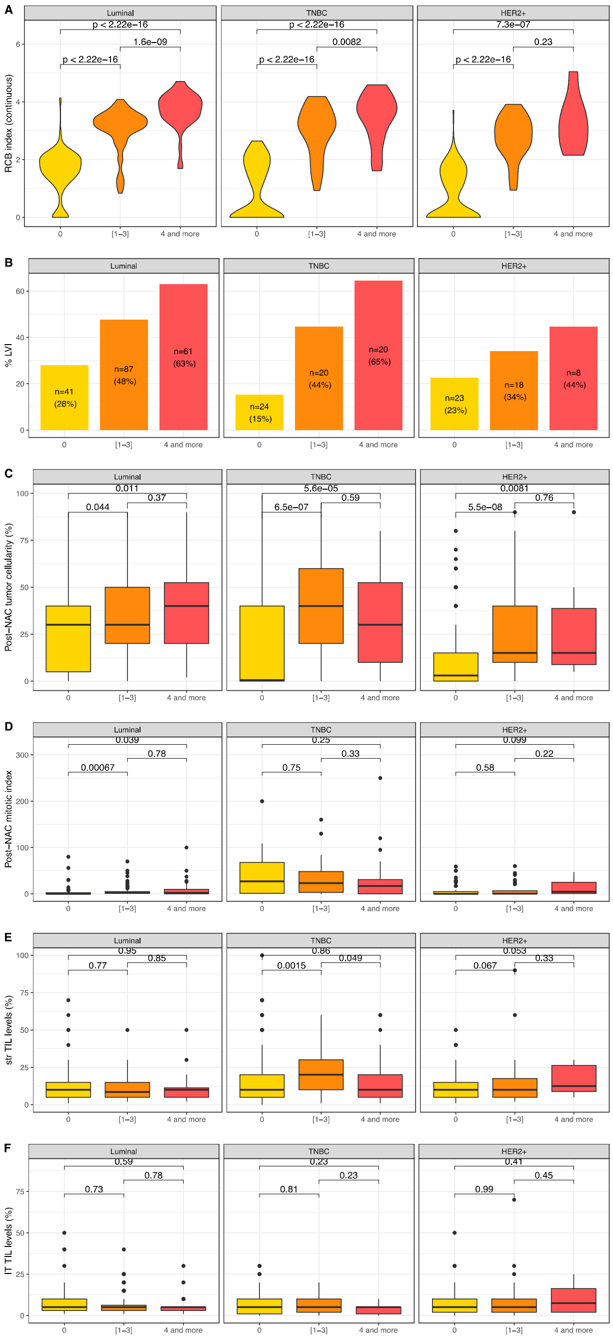

Figure S2. Association between post-NAC involvement and tumor characteristics according to BC subtype: RCB index (A); Lymphovascular invasion (B); Tumor cellularity (C); Post-NAC mitotic index (D); Stromal TIL levels (E). Intra tumoral (IT) TIL levels (F).

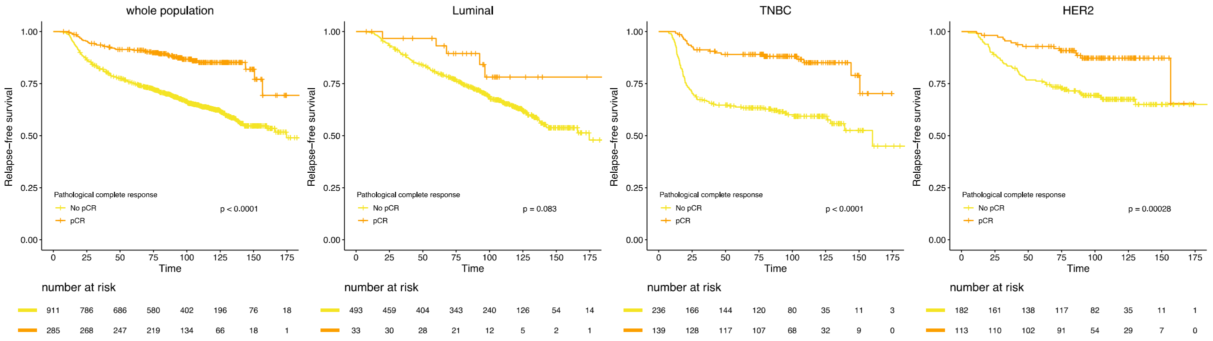

Figure S3. Relapse Free Survival (RFS) according to pCR after neoadjuvant chemotherapy in the whole population and according to breast cancer subtype.

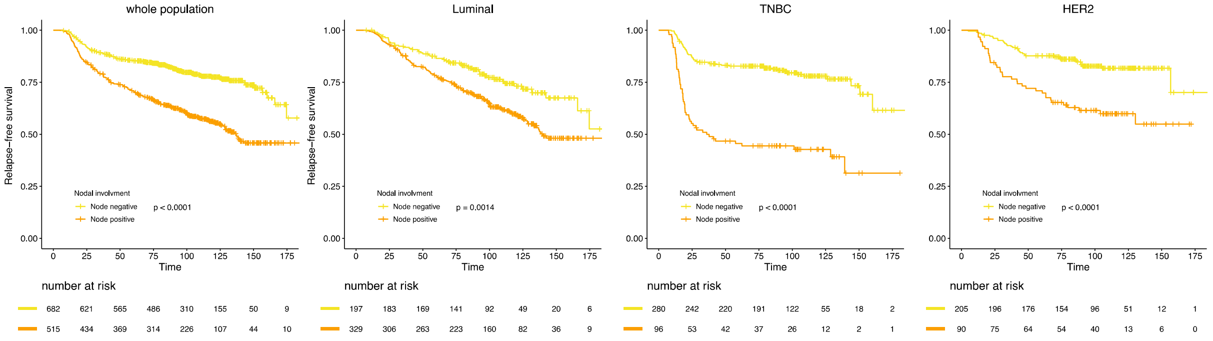

Figure S4. Relapse Free Survival (RFS) according to nodal status after neoadjuvant chemotherapy in the whole population and according to breast cancer subtype.
